# Differences in Serum Magnesium Levels in Diabetic and Non-Diabetic Patients Following One-Anastomosis Gastric Bypass

**DOI:** 10.3390/nu11091984

**Published:** 2019-08-22

**Authors:** Eva Winzer, Igor Grabovac, Bernhard Ludvik, Renate Kruschitz, Karin Schindler, Gerhard Prager, Carmen Klammer, Lee Smith, Friedrich Hoppichler, Rodrig Marculescu, Maria Wakolbinger

**Affiliations:** 1 Department of Social and Preventive Medicine, Centre for Public Health, Medical University of Vienna, Spitalgasse 23, 1090 Vienna, Austria; 2 Department of Medicine 1 and Karl Landsteiner Institute for Obesity and Metabolic Disorders, Rudolfstiftung Hospital, Juchgasse 25, 1030 Vienna, Austria; 3 Division of Internal Medicine, General Public Hospital of the Order of Saint Elisabeth, Völkermarkter Straße 15-19, 9020 Klagenfurt, Austria; 4 Division of Endocrinology and Metabolism, Department of Internal Medicine III, Medical University of Vienna, Spitalgasse 23, 1090 Vienna, Austria; 5 Division of General Surgery, Department of Surgery, Medical University of Vienna, Spitalgasse 23, 1090 Vienna, Austria; 6 Department of Internal Medicine, Convent of the Brothers of Saint John of God, Seilerstätte 2, 4021 Linz, Austria; 7 Cambridge Centre for Sport and Exercise Sciences, Anglia Ruskin University, Cambridge CB1 1PT, UK; 8 Special Institute for Preventive Cardiology and Nutrition—SIPCAN, Rabenfleckweg 8, 5061 Salzburg, Austria; 9 Division of Internal Medicine, Krankenhaus der Barmherzigen Brüder, Kajetanerplatz 1, 5010 Salzburg, Austria; 10 Clinical Institute for Medical and Chemical Laboratory Diagnostics, Department of Laboratory Medicine, Medical University of Vienna, Spitalgasse 23, 1090 Vienna, Austria

**Keywords:** serum magnesium, obesity, type 2 diabetes mellitus, one anastomosis gastric bypass

## Abstract

Patients with obesity and type 2 diabetes mellitus (T2DM) are regarded to have reduced serum magnesium (Mg) concentrations. We aimed to assess the changes in serum Mg concentrations at 12-month follow-up in patients, with and without T2DM, who underwent one anastomosis gastric bypass surgery. Overall, 50 patients (80% female, age 42.2 (12.5) years) with morbid obesity (mean baseline BMI 43.8 (4.3) kg/m^2^) were included in the analysis. Half of the included patients had T2DM diagnosed at baseline, and these patients showed lower serum Mg concentration (0.78 (0.07)) vs. 0.83 (0.05) mmol/L; *p* = 0.006), higher blood glucose levels (129.9 (41.3) vs. 87.6 (8.1) mg/dL; *p* < 0.001) as well as HbA1c concentrations (6.7 (1.4) vs. 5.3 (0.5)%; *p* < 0.001). During follow-up, BMI and glucose levels showed a decrease; however, serum Mg levels remained stable. At baseline 42% of patients were found to be Mg deficient, which was reduced to 33% at six months and to 30% at 12 months follow-up. Moreover, patients with T2DM had an odds ratio of 9.5 (95% CI = 3.0–29.7; *p* < 0.001) for magnesium deficiency when compared to patients without T2DM. Further research into the role of Mg and its role in T2DM and other obesity-related comorbidities are needed.

## 1. Introduction

Magnesium (Mg) is the second most abundant intracellular electrolyte serving as a co-factor in over 300 different enzymatic and biochemical reactions responsible for normal functioning of the organism [1,2,3]. It is also involved in blood glucose control, given its vital role in activating the beta-subunit of the insulin receptors [4]. However, taking into account the multitude of processes in which Mg is involved with, the physiological homeostasis of Mg is still not well understood [4,5].

Mg is present in both animal and plant based foods, with legumes, nuts, grains, seeds, and green leafy vegetables presenting the richest sources [6]. However, even with the relatively broad distribution of foods containing Mg, studies have indicated that intake of Mg is suboptimal, i.e., not reaching the recommended daily intake [7]. An analysis of nationally representative data from the United States showed that more than 80% of Americans do not meet the recommended daily intake with older African Americans showing the lowest Mg intake compared to other ethnic groups [8].

Reduced serum Mg (hypomagnesemia) has also been reported in patients with obesity, diabetes, and hypertension, and has been suggested as a biomarker for the symptoms cascade leading to the metabolic syndrome [9,10]. Studies reported that men with low serum Mg values had a two-fold increase in the incidence of type 2 diabetes mellitus (T2DM), while those in the highest quintile of Mg intake can reduce their diabetes risk by more than 30%. The exact mechanism by which hypomagnesemia induces or worsens T2DM is unclear, however, both secretion and action of insulin seem to be affected [11]. A possible mechanism could be the decreased renal Mg reabsorption as a consequence of insulin deficiency [11] or concomitant use of medications such as proton pump inhibitors, diuretics, statins, and antimicrobial agents [12].

The increasing disease burden of obesity and the increase in its worldwide prevalence presents a major public health issue [13]. The Austrian Health Interview Survey (AT-HIS) in 2014 included adults from a representative sample of the Austrian population. Overall, 15,771 individuals were interviewed and 56% were female. In this sample, 33% of the adults were overweighed (male 40%, female 26%) and 14% were obese (male 16%, female 13%) [14]. Consecutively, lasting therapeutic effects can only be achieved through weight loss, as it is associated with improved metabolic function as well as decrease in risk of comorbidities [15,16]. Weight loss can be achieved through various lifestyle interventions, such as increasing physical activity levels and controlling caloric intake. However, in morbidly obese patients bariatric surgery is preferred since it is associated with long term weight loss and reduced prevalence of comorbidities [17]. According to the 4th Global Registry Report of the International Federation for the Surgery of Obesity and Metabolic Disorders (IFSO) from 2018, 1255 bariatric surgeries have been reported in Austria between 2014–2018, and more than third preformed were the one anastomosis gastric bypass and mini-gastric bypass, making them a group of most commonly done bariatric surgeries in Austria [18].

Studies on obese patients following weight loss have reported inconsistent results, with some finding improvements in serum Mg levels and some reporting no changes in diabetic and non-diabetic patients with obesity [5,19,20]. As Mg has been linked to a myriad of positive health outcomes [21], and in light of rising trends in global obesity prevalence, our study aimed to investigate changes in serum Mg levels in patients with and without diabetes mellitus who underwent one-anastomosis gastric bypass (OAGB) before and after a 12-month follow-up. To our knowledge, this is the first study looking into Mg serum concentration of a patient cohort after this type of bariatric surgery.

## 2. Materials and Methods

### 2.1. Study Design

This study is a secondary analysis of the data and blood samples of participants who had been enrolled in the LOAD-study (“Link between Obesity And Vitamin D”), a six-months double-blind, placebo-controlled, randomized trial of vitamin D supplementation. The primary aim of the LOAD-study was to examine the efficacy and safety of a forced vitamin D regimen versus conventional supplementation on parameters of vitamin D metabolism in morbidly obese, vitamin D-deficient patients undergoing OAGB [22]. Inclusion criteria were; planned one-anastomosis gastric bypass (OAGB) surgery, above 18 years old, 25-hydroxy-vitamin D (25(OH)D) serum concentrations of below 75 nmol/L, no vitamin D supplementation, and a body weight below 140 kg (due to body weight limitation of the dual energy X-ray absorptiometry). Specific exclusion criteria included any other planned form of bariatric surgery than OAGB, hypo- and hypercalcemia, renal insufficiency, or primary hyperparathyroidism. In the first six months after OAGB surgery, the intervention group received the following vitamin D dosing regimen: Up to three oral loading doses of each 100,000 IU in the first month postoperatively, followed by maintenance dose of 3420 IU/day; and the control group received conventional supplementation (placebo in the first month with following maintenance doses of 3420 IU/day). Afterwards, both groups were recommended to continue the vitamin D_3_ supplementation until the follow-up visit at 12 months. The details on design, the used materials and methods, as well as the sample size calculation of the LOAD-study, have been previously published [23]. Primary analysis included the repeated-measures analysis of covariance (ANCOVA) using random error (linear mixed model) to assess the effects of time and the interaction for changes in parameters between the groups by using different covariance structure models as appropriate, and were adjusted for age, sex, and baseline values.

All OAGB procedures were performed at the General Hospital Vienna, Medical University of Vienna by the same surgical team using a laparoscopic approach. It is a simplified procedure that consists of a unique gastrojejunal anastomosis between a 30 to 40 mL sleeve gastric pouch and a jejunal omega-loop of approximately 200 cm [24]. The study methods are in accordance with the CONSORT (Consolidated Standards Of Reporting Trials) guidelines for reporting randomized trials [25].

This study was approved by the local Ethics Committee of the Medical University of Vienna (No° 1899/2013), by the Austrian Competent Authority (No° LCM-718280-0001), registered at clinicaltrials.gov (Identifier: NCT02092376) and EudraCT (Identifier: 2013-003546-16), complies with the Declaration of Helsinki [26], and conducted from April 2014 to June 2016 at the Medical University of Vienna. The study participants were scheduled for OAGB surgery and all of them gave written informed consent preoperatively.

### 2.2. Assessment of Variables

Data were assessed before surgery (T0), at 6 months (T6), and at 12 months postoperatively (T12). At T0, age, sex, and medical history (e.g., comorbidities, prescribed medication) were collected as previously described [23]. Height and body weight (measured with the calibrated scale seca mBCA 515) were obtained for each participant at the three time points.

Blood samples were collected and serum Mg, glucose, HbA_1c_, triglycerides, total cholesterol, HDL cholesterol, and LDL cholesterol were used for this secondary analysis.

### 2.3. Statistical Analysis

The results are expressed as mean (standard deviation) for continuous and as percentages for categorical variables. In order to test for normal distribution, a visual test (histograms and box plots) was used and the Kolmogorov-Smirnov test was applied in addition.

At baseline the difference between diabetic patients and parameters such as sex, age, BMI, glucose, HbA_1c_, oral antidiabetic drugs (OAD) and insulin use, serum lipid levels such as triglycerides, total cholesterol, HDL cholesterol, cholesterol/HDL ratio, and LDL cholesterol was assessed with *t*-test and Chi^2^-test. The statistical association between serum Mg and parameters such as diabetes mellitus, glucose, HbA1c, and serum lipid levels was examined by Pearson correlation coefficients.

The main outcome of interest was the change in serum Mg concentration in the first postoperative year. Moreover, the changes in BMI, blood sugar levels, and serum lipid levels were also assessed. We used the entire sample for this analysis. We applied repeated-measures analysis of covariance (ANCOVA) using random error (linear mixed model) to assess the effect of time by using different covariance structure models as appropriate and which were adjusted for age, sex, and baseline values to supply an unbiased estimate of the mean difference [27]. A post hoc analysis with Bonferroni correction was used.

Estimates of the prevalence of Mg deficiency over time were calculated using generalized estimating equation (GEE) with a logit link function for binary outcomes and unstructured covariance matrices. With this approach, we examined effects with time as repeated factor with Mg deficiency as dependent variable, adjusted for age and sex.

Means were compared unadjusted without imputation of missing data. All statistical analyses were performed with IBM^®^ SPSS^®^ Statistics for Windows, Version 23 software (IBM Corporation, Armonk, NY, USA). *p*-values < 0.05 were considered statistically significant and all tests were two-sided.

## 3. Results

### 3.1. Patients’ Characteristics

Out of 67 eligible patients, 17 declined to participate (25%). The remaining 50 patients entered the randomized controlled trial [22]. Baseline characteristics are demonstrated in Table 1. Fifty percent of the patients suffered from type 2 diabetes mellitus (T2DM) and 22% of the patients used statins or fibrates. Diabetic patients and patients using statins/fibrates were significantly older. Two-thirds of the diabetic patients and over 90% of patients using statins/fibrates had an Mg deficiency. Serum lipid levels were not significant between diabetic and non-diabetic patients.

There was a statistically significant association between serum Mg and T2DM (r = −0.38, *p* = 0.006), glucose (r = −0.42, *p* = 0.002), HbA_1c_ (r = −0.52, *p* < 0.001) and statin/fibrate use (r = −0.48, *p* < 0.001). There was no statistically significant association between serum Mg and serum lipid levels.

### 3.2. Associations between Diabetic Patients and Serum Magnesium, Blood Glucose and Serum Lipid Parameters in the First Postoperative Year

As this study is a secondary analysis of the LOAD-study, a post hoc power calculation was applied for the main outcome (change in serum Mg concentration). This power analysis showed that the current study had a power of 83% (α = 0.05) to detect differences in the mean serum Mg concentration between non-diabetics (*n* = 25) and diabetics (*n* = 25) after 12 months.

Table 2 shows the comparison between diabetic and non-diabetic patients on BMI, serum Mg, blood glucose, and serum lipid values over time. At T0, diabetic patients showed significantly lower serum Mg, and higher glucose and HbA_1c_ concentrations. At T12, significant group differences in BMI and glucose values could be found. There were also between group differences in serum Mg concentration at T6 and at T12. In addition, BMI, HbA_1c,_ and triglyceride values showed a significant group and time interaction, with higher concentrations in diabetic patients.

Moreover, diabetic patients had an odds ratio of 9.5 (95% CI = 3.0, 29.7; *p* < 0.001) for Mg deficiency compared with non-diabetics, adjusted for age and sex. In total, Mg deficiency was found in 33% of the patients at T6 and 30% at T12.

## 4. Discussion

In our study of a cohort of patients with morbid obesity that underwent OAGB surgery, we found significantly lower serum Mg levels in patients with vs. without T2DM at baseline. This finding is in line with other studies confirming the association of low serum Mg and the presence of T2DM [5,19,20]. Insulin resistance and deficiency are considered to inhibit the reabsorption of Mg and accelerate its excretion through the kidneys [28]. Low levels of Mg in turn further decrease insulin sensitivity and the function of insulin receptors [29]. Overall, our patients with T2DM had an 8.5-fold greater risk of being Mg deficient compared to patients without T2DM.

Regarding differences in Mg intake being the reason for low serum levels, a cross-sectional study in a Chinese population reported no significant associations between dietary Mg intake (evaluated using a semi-quantitative food frequency questionnaire) and T2DM prevalence [20]. Moreover, there was no correlation between dietary Mg intake and serum concentrations in participants with diabetes. These results are similar to those of other studies emerging from Asia. In contrast, some investigators reported a significantly inverse correlation between dietary Mg and diabetes [30,31,32]. This may be due to difference in the degree of insulin resistance in the Asian population in comparison to Western population [33]. Further differences may be found in the dietary patterns, as a study using US nationally representative data found differences among different ethnic groups and their overall Mg intake [8]. However, this study did not specifically investigate the Asian population in the US due to a lack of data. These results are contradicted by a recent umbrella review of observational and intervention studies indicating that, out of 55 independent outcomes associated with Mg levels, there was highly suggestive evidence that higher Mg intake at baseline was associated with lower T2DM incidence [21]. The authors of this umbrella review acknowledge that there is a possible bias in observational studies, since higher Mg intake might be associated with a healthier lifestyle and dietary patterns. There is also a rather weak evidence that Mg supplementation is able to significantly improve the results of the 2 h oral glucose tolerance test in high risk groups for T2DM. Review studies indicated that more randomized controlled trials focusing on different age groups, as well as factors like metabolic control and baseline Mg, are needed in order to provide recommendations on the supplementation of Mg in patients with diabetes [34].

After OAGB surgery, there was a continuous drop in BMI in all patients, which was significantly greater in nondiabetic subjects at 6 and 12 months follow-up. Differences remained significant after adjusting for age, gender, and baseline values, and showed a significant group-time interaction. However, the drop in BMI was not followed by changes in the serum Mg concentrations. Both groups showed no changes in mean concentrations of serum Mg throughout the study. A 2009 study by Johansson et al. examined 21 patients without diabetes after Roux-en-Y gastric bypass surgery [35]. They reported lower plasma glucose and increased serum Mg levels. Similarly, a 2019 study by Mikalsen Meyer et al. [19] showed that a modest weight loss of about ten kilograms in obese patients with lifestyle interventions already increased serum Mg levels in obese patients with and without T2DM by around 5%. After Roux-en-Y gastric bypass surgery, the serum Mg concentrations continued to rise in patients with T2DM and remained stable in non-diabetic patients [19].

Some differences for the controversial findings may arise from surgical techniques, as different techniques are associated with different effects on serum Mg concentrations. An analysis of patients after Roux-en-Y gastric bypass surgery found that 32% of patients were Mg deficient. The results showed a mild increase in Mg levels following surgery after two years follow-up and no changes in mean values of serum Mg concentration at time of the analysis (in mean almost seven years after intervention) [36]. These results are similar as we also found 33% and 30% at 6 and 12 months after surgery, respectively.

This secondary analysis is limited by a rather small sample size that was based on sample size-calculations taking into consideration serum differences of vitamin D concentration. However, as we show significant differences, the sample size calculation can be considered valid. The study is comprised of over 80% women, which is expected in a patient population undergoing bariatric surgery, but prohibits generalizations to male patients. Moreover, there is no gold standard in determining Mg status. The strengths of the study include detailed pre- and post-operative data of patients undertaking OAGB, a relatively new surgical procedure that has not been evaluated in regards to Mg serum concentrations.

## 5. Conclusions

In our patients with obesity, we observed significantly lower serum Mg levels in patients with vs. those without T2DM. Following bariatric surgery and weight loss, Mg serum concentrations remained stable. Since low Mg levels are associated with hypertension, oxidative stress, and coronary heart disease [9], it is possible that normalized Mg levels could reduce the risk of comorbidities associated with obesity and T2DM, which, however, needs to be proven.

## Figures and Tables

**Table 1:** Patients’ characteristics.

Parameters	Total	Type 2 Diabetes Mellitus		*p*-Value ^+^
		no (*n* = 25)	yes (*n* = 25)	
	Mean ± SD Number (%)	Mean ± SD Number (%)	Mean ± SD Number (%)	
Female	40 (80)	19 (76)	21 (84)	0.480
Age (years)	42.2 ± 12.5	35.6 ± 11.9	48.8 ± 9.3	**<0.001**
BMI (kg/m^2^)	43.8 ± 4.3	43.7 ± 4.9	43.9 ± 3.7	0.835
Supplementation				
Vitamin D	8 (16)	3 (12)	5 (20)	0.440
Iron	1 (2)	0 (0)	1 (4)	0.312
Diuretics use	12 (24)	2 (8)	10 (40)	**0.008**
Magnesium (mmol/L)	0.81 ± 0.07	0.83 ± 0.05	0.78 ± 0.07	**0.006**
Deficient (<0.8 mmol/L)	21 (42)	5 (20)	16 (64)	**0.002**
Glucose (mg/dL)	108.7 ± 36.4	87.6 ± 8.1	129.9 ± 41.3	**<0.001**
HbA_1c_ (%)	6.0 ± 1.3	5.3 ± 0.5	6.7 ± 1.4	**<0.001**
OAD use	9 (18)	0 (0)	9 (36)	**0.001**
Insulin use	6 (12)	0 (0)	6 (24)	**0.009**
Triglycerides (mg/dL)	155.8 ± 79.8	164.5 ± 89.4	147.2 ± 69.6	0.447
Total cholesterol (mg/dL)	198.2 ± 46.9	197.6 ± 31.9	198.9 ± 59	0.920
HDL cholesterol (mg/dL)	47.3 ± 12.3	46.3 ± 10.3	48.2 ± 14.1	0.578
Cholesterol/HDL ratio	4.4 ± 1.2	4.5 ± 1.3	4.3 ± 1.2	0.573
LDL cholesterol (mg/dL)	120.6 ± 40.3	120 ± 29.9	121.2 ± 48.9	0.916

Body Mass Index (BMI), Hemoglobin A1c (HbA_1c_), Oral antidiabetic drugs (OAD), High density lipoprotein (HDL), Low density lipoprotein (LDL), ^+^ *T*-test or Chi^2^-test. A *p*-value in bold denotes a significant difference (*p* < 0.05).

**Table 2:** Change in BMI, serum magnesium, glucose, and lipid parameters over time in diabetic and non-diabetic patients.

Parameters		Baseline	*p*-Value ^+^	6 Months	*p*-Value ^+^	12 Months	*p*-Value ^+^	*p*-Value Group ^+^	*p*-Value Time ^+^	*p*-Value Group * Time ^+^
		Mean ± SD		Mean ± SD		Mean ± SD				
BMI (kg/m^2^)	Non-diabetics	43.7 ± 4.9	0.504	32.0 ± 3.8	**0.020**	28.0 ± 4.2	**0.001**	**0.004**	**<0.001**	**0.001**
	Diabetics	43.9 ± 3.7		32.4 ± 3.9		29.0 ± 2.5				
Magnesium (mmol/L)	Non-diabetics	0.83 ± 0.05	**0.006**	0.84 ± 0.04	0.350	0.84 ± 0.05	0.248	**0.033**	0.286	0.474
	Diabetics	0.78 ± 0.07		0.78 ± 0.05		0.78 ± 0.09				
Glucose (mg/dL)	Non-diabetics	87.6 ± 8.1	**<0.001**	83.5 ± 5.5	**0.029**	82.2 ± 5.8	**0.019**	**<0.001**	**0.001**	0.733
	Diabetics	129.9 ± 41.3		134.6 ± 26.5		131 ± 25.7				
HbA1c (%)	Non-diabetics	5.3 ± 0.5	**0.001**	5.1 ± 0.3	**0.006**	5.1 ± 0.4	0.057	0.264	**<0.001**	**<0.001**
	Diabetics	6.7 ± 1.4		6.2 ± 0.7		6.4 ± 0.8				
Triglycerides (mg/dL)	Non-diabetics	164.5 ± 89.4	0.072	91.3 ± 29.8	0.018	85.3 ± 34.9	0.643	0.453	**<0.001**	**0.007**
	Diabetics	147.2 ± 69.6		144.2 ± 30.6		104.6 ± 23.2				
Total cholesterol (mg/dL)	Non-diabetics	197.6 ± 31.9	0.707	165.5 ± 38.7	0.507	163.7 ± 29.6	0.474	0.547	**<0.001**	0.604
	Diabetics	198.9 ± 59		170.5 ± 38.9		169.5 ± 56.1				
HDL cholesterol (mg/dL)	Non-diabetics	46.3 ± 10.3	0.324	47.4 ± 10.2	0.995	53.8 ± 9.4	0.303	0.301	**<0.001**	0.681
	Diabetics	48.2 ± 14.1		45.1 ± 8.9		55.1 ± 11.2				
Cholesterol/HDL ratio	Non-diabetics	4.5 ± 1.3	0.272	3.6 ± 0.8	0.761	3.1 ± 0.8	0.900	0.582	**<0.001**	0.726
	Diabetics	4.3 ± 1.2		3.7 ± 0.7		3.1 ± 1.0				
LDL cholesterol (mg/dL)	Non-diabetics	120 ± 29.9	0.849	99.8 ± 32.3	0.971	92.9 ± 24.9	0.735	0.924	**<0.001**	0.916
	Diabetics	121.2 ± 48.9		96.5 ± 35.8		93.5 ± 52.1				

Body Mass Index (BMI), Hemoglobin A1c (HbA_1c_), High density lipoprotein (HDL), Low density lipoprotein (LDL). ^+^ Repeated measure analysis of variance and post hoc analysis with Bonferroni correction, adjusted for baseline value, age, and sex. Group * Time means group-by-time interaction. A *p*-value in bold denotes a significant difference (*p* < 0.05).
